# Chemokine-Releasing Nanoparticles for Manipulation of the Lymph Node Microenvironment

**DOI:** 10.3390/nano5010298

**Published:** 2015-03-05

**Authors:** Taissia G. Popova, Allison Teunis, Ruben Magni, Alessandra Luchini, Virginia Espina, Lance A. Liotta, Serguei G. Popov

**Affiliations:** 1 Center for Applied Proteomics and Molecular Medicine, Department of Molecular Microbiology, School of Systems Biology, George Mason University, Manassas, VA 20110, USA; E-Mails: tpopova@gmu.edu (T.G.P.); ateunis@gmu.edu (A.T.); rmagni@gmu.edu (R.M.); aluchini@gmu.edu (A.L.); vespina@gmu.edu (V.E.); lliotta@gmu.edu (L.A.L.); 2 National Center for Biodefense and Infectious Diseases, Department of Molecular Microbiology, School of Systems Biology, George Mason University, Manassas, VA 20110, USA

**Keywords:** nanoparticles, chemokines, neutrophils, lymph node

## Abstract

Chemokines (CKs) secreted by the host cells into surrounding tissue establish concentration gradients directing the migration of leukocytes. We propose an *in vivo* CK gradient remodeling approach based on sustained release of CKs by the crosslinked poly(N-isopropylacrylamide) hydrogel open meshwork nano-particles (NPs) containing internal crosslinked dye affinity baits for a reversible CK binding and release. The sustained release is based on a new principle of affinity off-rate tuning. The NPs with Cibacron Blue F3G-A and Reactive Blue-4 baits demonstrated a low-micromolar affinity binding to IL-8, MIP-2, and MCP-1 with a half-life of several hours at 37 °C. The capacity of NPs loaded with IL-8 and MIP-1α to increase neutrophil recruitment to lymph nodes (LNs) was tested in mice after footpad injection. Fluorescently-labeled NPs used as tracers indicated the delivery into the sub-capsular compartment of draining LNs. The animals administered the CK-loaded NPs demonstrated a widening of the sub-capsular space and a strong LN influx of leukocytes, while mice injected with control NPs without CKs or bolus doses of soluble CKs alone showed only a marginal neutrophil response. This technology provides a new means to therapeutically direct or restore immune cell traffic, and can also be employed for simultaneous therapy delivery.

## 1. Introduction

Lymphoid organs and tissues of the host play key roles in the protection of the host from infections and spread of tumors. The immune response induced by microbial or tumor antigens involves a coordinated activity by a multitude of host cell populations. One of the important features of this response is a sophisticated process of direct leukocyte trafficking regulated in part by a chemotactic communication system based on interactions of chemokines (CKs) with cognate cellular receptors [1,2].

CKs secreted by the host cells into the surrounding tissue establish soluble and/or immobilized concentration gradients directing the migration of leukocytes toward areas of high CK concentration. Macrophages, dendritic cells (DCs) and neutrophils (PMNs) use chemotaxis for delivery and presentation of antigens to secondary lymphoid organs. Many pathogens have evolved sophisticated means to thwart the host defense system, including the blockade of leukocyte chemotaxis toward the infection [3,4]. Development of therapeutic means to restore and predictably manipulate the chemotaxis of immune cells is an attractive concept that can influence the design of new vaccine adjuvants, anti-tumor reagents, anti-inflammatory and anti-microbial treatments. A promising approach is to locally engineer CK gradients with the goal of promoting the accumulation of key immune players, such as DCs and neutrophils, or eliciting cascades of immune responses to eliminate pathogens or tumors [5,6,7].

Recent advances in nanotechnology now offer innovative ways to reach this goal using controlled-release materials to engineer CK gradients for basic studies and therapeutic applications [8,9,10,11,12]. It has been reported that biodegradable NPs providing a sustained release of various CKs are able to direct *in vitro* migration of dendritic cells (DCs), monocytes (DC precursors), and T cells [8,9]. In addition to immune cells, CK-releasing NPs have been used to recruit endogenous progenitor cells to local sites to promote wound healing, tissue regeneration [5,13], and restore perfusion in an ischemic tissue [14] with a potential to treat myocardial infarctions [15]. Importantly, sustained release of CK eliminated the need for repeated injections, a major advantage for clinical applications.

However, the loading of CKs into existing scaffolds suffers considerable limitations. Obtaining functional release of many CKs of interest is problematic, likely due to the harsh microenvironment within eroding particles, low capacity, and complex experimental procedures [12,16,17]. Thus, the need exists for an alternative system that could more efficiently entrap CKs with higher net bioactivity, and provide “point-source” release of a broader range of attractants to engineer CK gradients [17]. We propose to overcome these methodological limitations using a new multifunctional micro- and nano-particle (NP) platform technology recently invented by us [18,19,20,21,22,23,24,25,26,27,28].

The NPs we synthesized consist of the non-toxic polyacrylamide hydrogel scaffold demonstrating long-time persistence in tissues [29,30,31]. A variety of inner baits of different chemical nature can be quickly incorporated into the NPs to capture from the environment in one step a wide range of molecules including the CKs [19]. Among such baits the textile dyes of triazine, acidic, basic and disperse types attracted attention due to their affinity interactions with broad classes of protein ligands on the basis of specific molecular recognition processes [32,33]. The dye-ligand matrices used in affinity chromatography are thought to mimic the structural features of the corresponding natural substrates, cofactors, *etc.*, and are therefore considered pseudo-affinity matrices [34].

The NPs can be preloaded with the substances of interest which can be reversibly released from the baits at a controlled rate. The hydrogel structure protects the loaded cargo from degradation while the process of loading and release under mild physiological conditions assures preservation of its biological function. We have currently tested more than 20 different bait formulations ensuring capture and release of proteins [19,21]. Based on this platform, we propose a simple and versatile approach for creating CK-loaded NPs which could serve both as a research tool and a prototype of future immunotherapies for manipulating leukocyte trafficking. Our NPs are ideally suited as carriers for reversible loading and controlled release of CKs, which generally are cationic small proteins. The baits we use mimic the natural interactions of proteoglycans with CKs essential for *in vivo* activity. Our methodology can be applied beyond CKs to many host immunomodulatory proteins.

The main goal of this work was to demonstrate feasibility of the CK gradient remodeling approach using our NP technology to increase an influx of neutrophils into draining lymph nodes (LNs) of mice by the CK-releasing NPs. As a first step, we investigated the affinity of binding and dynamics of a sustained release of some CKs by NPs chosen to target an immune response. Secondly, we demonstrated quick NP delivery to draining lymph nodes (LNs) by subcutaneous injection of NP suspension into footpads of mice. Finally, the capacity of IL-8 and MIP-1α-loaded NP to increase neutrophil recruitment to LNs was tested after subcutaneous administration to mice. Our approach can be considered as a model of future therapeutic treatments targeting abnormalities in the recruitment of immune cells.

## 2. Results

### 2.1. NPs Containing Different Chemical Baits Can Be Loaded with CKs

The NPs we synthesized are based on poly(N-isopropylacrylamide) (pNIPAm) and methylenebisacrylamide as a cross-linker co-polymerized with allylamine or acrylic acid (AAc,) for incorporation of different chemical baits [19]. The NPs were characterized by their light scattering properties as described previously [19,21]. The average particle diameter in PBS at 25 °C for different batches was in the range of 600–700 nm with a standard deviation of size from 3 to 17 nm. The polydispersity index was found to be 0.2–0.4 indicating a low level of aggregation. These particles contain molecular pores of sufficient size to allow diffusion of the small proteins such as CKs (8–20 kDa) inside the particle core.

**Figure A1:**
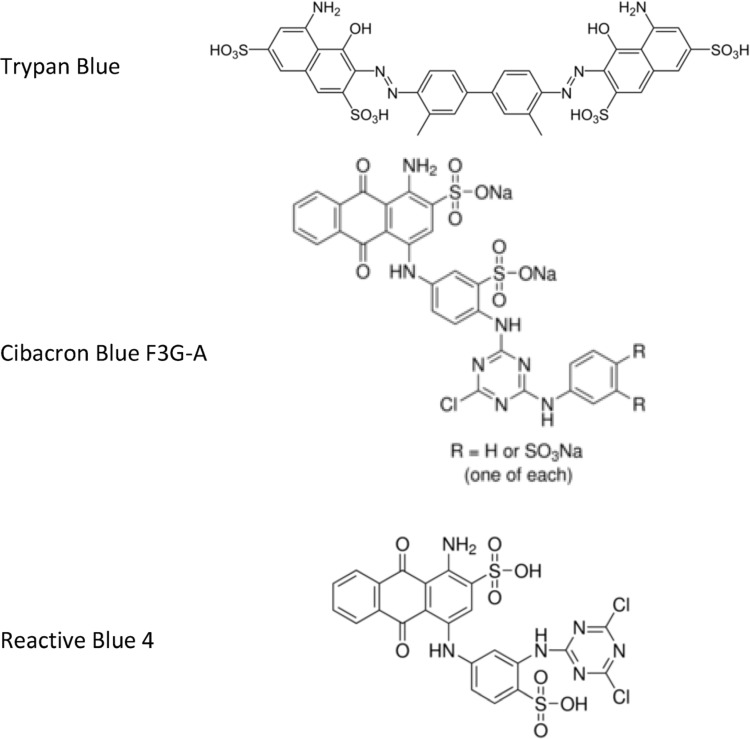
Chemical structures of the Trypan Blue, Cibacron Blue F3G-A (Cibacron), and Reactive Blue 4 (Reactive Blue) dyes.

As the baits we used the triazine dyes Cibacron Blue F3G-A (Cibacron) and Reactive Blue 4 (Reactive Blue), which were chemically coupled through the amino group of allylamine-containing NPs [33]. The toluidine dye Trypan Blue was coupled to the carboxyl groups of the AAc-co-polymerized NPs activated by the water-soluble carbodiimide [27]. The chemical structures of the dyes containing multiple aromatic, condensed, and heterocyclic rings, as well as 2–4 negatively-charged sulfate groups per molecule, are shown in Figure A1. Overall, the dyes are capable of electrostatic, hydrophobic, and hydrogen-bonding interactions with the proteins [32,33,34].

**Figure A2:**
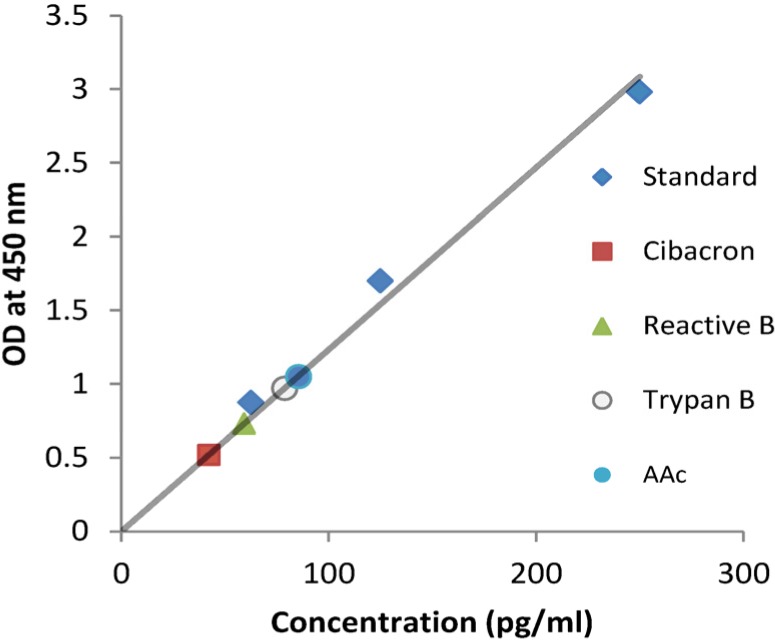
ELISA of IL-8 binding (250 pg/mL) to NPs containing different baits. The particle suspension (5% wet *v*/*v*) of the NPs was incubated with IL-8 for 30 min at room temperature. Diamonds correspond to the standard IL-8 concentrations used to draw a linear calibration plot.

The bait-coupled NPs were tested for their binding with IL-8. Since the AAc-containing NPs carry a polyelectrolytic negative charge in physiological conditions, these particles were also included in the test. Taking into account that the CK-binding capacity of the NPs can be influenced by pH [18], all experiments were carried out in PBS at the slightly alkaline pH 7.4 of lymph and blood. The 5% particle suspension was incubated with the CK in PBS at room temperature and the amount of bound CK was tested using sandwich ELISA. The CK experiments are often reported to be complicated by protein aggregation, which was minimized using freshly diluted stock solutions and low CK concentration (250 pg/mL) within the analytical interval of our ELISA test. The NPs demonstrated a range of affinities which allowed extraction of 66%, 68%, 76% and 84% of CK being present in solution by the AAc-, Trypan Blue-, Reactive Blue- and Cibacron-containing NPs, correspondingly (Figure A2). The Cibacron and Reactive Blue NPs were chosen for further experiments because of their superior binding.

**Figure 1:**
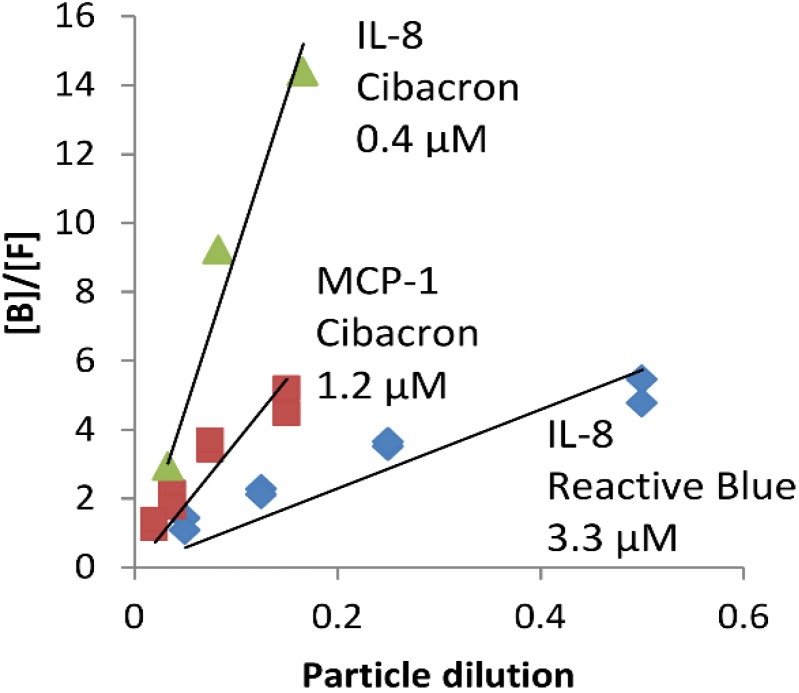
Binding isotherms of IL-8 (0.25 ng/mL) and MCP-1 (1 ng/mL) in PBS at 4 °C, 18 h with different concentrations of NPs (5% wet volume suspension diluted as indicated). Triangles and squares correspond to Cibacron, and diamonds to Reactive Blue. [F] and [B] stand for concentrations of free and particle-bound CKs, respectively.

To demonstrate the dynamic equilibrium nature of CK–bait interactions required for the release of CKs from NPs *in vivo*, we tested the binding of the CKs at different concentrations of NPs. In these experiments, we followed the procedure used later for the preparation of CK-loaded NPs for animal challenge. IL-8 and MCP-1 (at 250–500 pg/mL) were loaded onto Cibacron and Reactive Blue NPs at 4 °C overnight, quickly spun at room temperature to pellet the NPs after the loading step, and the amount of free CK in supernatants (Sups) was measured with ELISA. The degree of binding depended on the amount of particles interacting with CK, in general agreement with the mass law-driven equilibrium binding (Figure 1). The binding curves, however, somewhat deviated from the expected straight lines, indicating a certain degree of heterogeneity of the binding sites.

The dissociation equilibrium constants (*K*_d_) were calculated from the slopes of the straight lines as an approximate measure of affinity using the following equation, assuming independent CK binding to equal binding sites on the NPs:

*K*_d_ = [F][P]/[B]

where F and B stand for free and particle-bound CKs, correspondingly. P refers to the CK binding sites on the NPs.

In the case of the high-capacity NPs (estimated in our case to be close to 4 × 10^−5^ M based on the amount of dye used for coupling; see Materials and Methods), binding of CK at the concentrations used in our experiments (below 1 μg/mL or 10^−7^ M) does not change [P] to any considerable extent. Therefore, [P] = α*P*_0_, where *P*_0_ is the total concentration of binding sites, and α reflects a dilution of the stock NP suspension upon mixing with CK solution. The values of *K*_d_ calculated from the slopes of the plots in Figure 1 were found to be in the low-micromolar to high-nanomolar range favorable for the sustained CK release.

**Figure A3:**
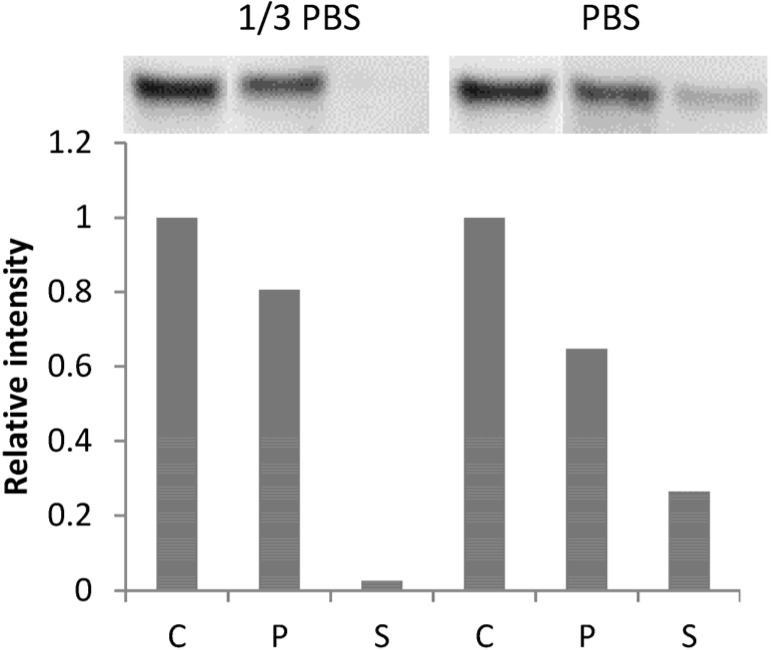
Binding of MIP-2 (200 ng/mL) with Reactive Blue NP suspension (10% wet *v*/*v*) in PBS and three-fold diluted PBS at 4 °C overnight. After incubation, the particles were quickly pelleted and the amount of CK determined by Western blot. Upper panels, the Western blot images; lower panels, band intensities corresponding to the control amount of CK before binding to the NPs (C), NP pellet (P), and Sups (S).

Although the above experiments demonstrated the binding affinity of CKs to bait-containing NPs in diluted solutions (<1 ng/mL), the experiments *in vivo* may require a delivery of small volumes of NPs containing high concentrations of CKs (up to 1 μg/mL [35,36]). Therefore, we tested the extent of binding using Western blot at CK levels of 0.2–2 μg/mL. To reach higher binding capacity, the CK loading onto NPs was carried out in the three-fold diluted PBS (1/3 PBS) which was expected to increase affinity due to reduced shielding of electrostatic interactions by the buffer ions. As a representative example, Figure A3 shows that the MIP-2 binding with Reactive Blue NPs at the decreased ionic strength of the buffer led to a virtually complete removal of the CK from solution. Similar results were obtained with Cibacron NPs (not shown). However, the amount of CK released from the NPs in Figure A3 remained lower than the input amount in control wells. It is likely that due to the CK self-aggregation, a small fraction of the CK was present in the dimeric or multimeric form and therefore was not accounted for. It is also possible that a portion of CK remained tightly bound to the NPs.

### 2.2. CK-Loaded NPs Provide a Sustained Release of Their Cargo

**Figure 2:**
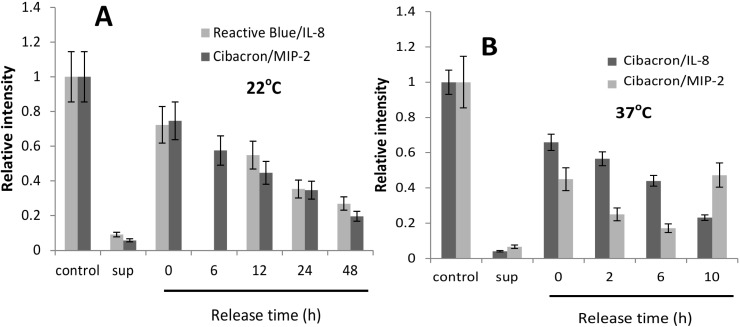
Release of IL-8 and MIP-2 from reactive Blue and Cibacron NPs. CKs (2 μg/mL) were mixed with indicated NPs (10% *v*/*v* suspension) in 100 μL of three-fold diluted PBS (1/3 PBS) and incubated at 4 °C overnight. After incubation the NPs were pelleted for 5 min at 16,000 g and room temperature, re-suspended in 1 mL of 1/3 PBS, and incubated at 22 °C (**A**) or 37 °C (**B**). Portions of the suspension were withdrawn at indicated times, the NPs were pelleted for 5 min at 16,000 *g*, and supernatants removed. The remaining pellets were boiled for 5 min in the SDS loading buffer and the amount of CK in solution determined by Western blot. In (**B**) the 1/3 PBS buffer was supplemented with 1 mg/mL BSA. The experiments were run in duplicate. Error bars indicate SD of relative band intensities for pairwise measurements (*n* = 6).

**Figure A4:**
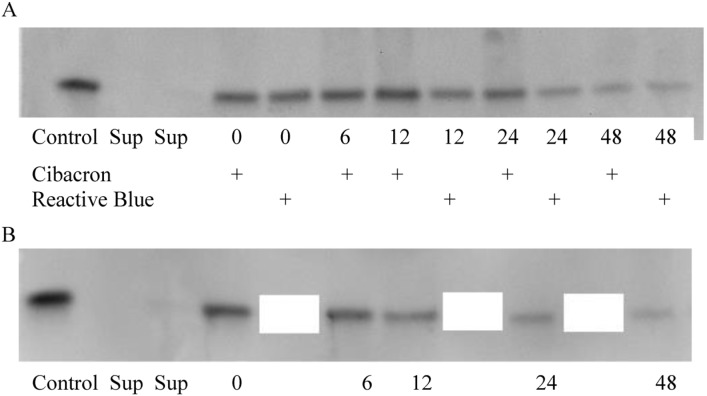

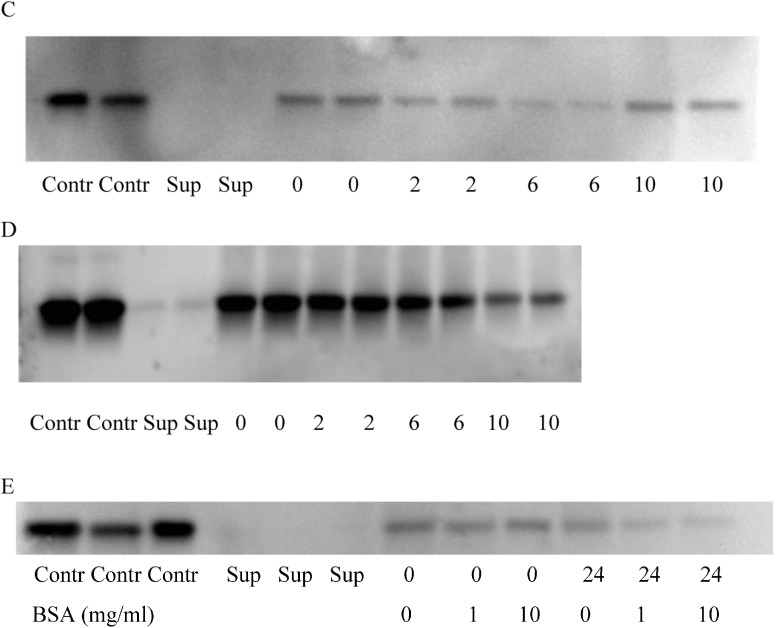
Raw data illustrating western blots in the experiments presented in Figure 2 and Figure 3. (**A**) IL-8 release from Cibacron and Reactive Blue NPs, no BSA, 22 °C; (**B**) MIP-2, Cibacron NPs, no BSA, 22 °C; (**C**) MIP-2 release from Cibacron NPs, no BSA, 37 °C; (**D**) IL-8 release from Cibacron NPs, no BSA, 37 °C; (**E**) MIP-2 release from Cibacron NPs, with BSA, 22 °C. Numbers in the legends indicate time (h) after initiation of dissociation.

To test the CK release rates the NPs were loaded with CKs in 1/3 PBS at 4 °C overnight to ensure maximum binding and then quickly pelleted to remove supernatants (Sups). The NPs were then re-suspended in a much larger volume of buffer (compared to the loading step) to initiate the CK dissociation, and the suspension was incubated at the indicated temperature (22 °C or 37 °C) with slight agitation. The NPs from the aliquots of this suspension corresponding to a certain fraction of the total volume were pelleted and the amount of bound CK was determined by Western blot with a CK-specific antibody. An equal fraction of the total CK amount used for loading served as a control representing the ideal case of 100% binding and release. The intensities of the bands at different time points (Figure A4) were compared with the control band intensity. Typical time courses of the IL-8 and MIP-2 release by Cibacron and Reactive Blue NPs are shown in Figure 2. It was found that after loading at 4 °C almost all amounts of CKs were extracted from solution by the NPs. However, a re-suspension of the NP pellet in the fresh buffer released a portion of the CK which appeared to be loosely bound. It was evident from the comparison between the amount of CK in the control and on the NPs immediately after the re-suspension. However, the rest of the CK displayed an expected gradual release rate. Another caveat in these experiments was an observed increase of the MIP-2 amount in the NP pellet after the 10-h incubation at 37 °C. This effect which might reflect the aggregation of the dissociated CK and its co-precipitation with NPs during centrifugation was not studied further. An approximation of the release kinetic with the first-order rate equation was used to estimate the dissociation constants (*k*_d_) and half-lives (ln2/*k*_d_) of the bound CKs (Table 1). According to these data, at the physiological temperature of 37 °C the release of both CKs is expected to take place for more than 20 h (corresponding to ~5× half-life times). The activation energies of the dissociation process calculated from the Table 1 data for IL-8 and MIP-2 were found to be close to each other (70 and 68 kJ/mol, respectively).

**Table 1:** Kinetic dissociation constants and half-dissociation times in PBS of IL-8 and MIP-2 bound to Cibacron (CB) and Reactive Blue (RB) NPs.

Bait	22 °C		37 °C	
	IL-8	MIP-2	IL-8	MIP-2
CB	*k*_d_ 0.016 ± 0.005 * h^−1^ *t*_1/2_ 43.5 h	*k*_d_ 0.030 ± 0.002 * h^−1^ *t*_1/2_ 23.7 h	*k*_d_ 0.094 ± 0.017 * h^−1^ *t*_1/2_ 7.44 h (BSA 1 mg/mL) ***	*k*_d_ 0.17 ± 0.05 * h^−1^ *t*_1/2_ 4.1 h (BSA 1 mg/mL) ***
RB	*k*_d_ 0.022 ± 0.004 * h^−1^ *t*_1/2_ 31.3 h	ND **	ND **	ND **

Notes: * Standard deviations of *k*_d_ calculated from the linear approximations of the kinetic curves; ** Not determined; *** BSA was included in the dissociation buffer.

### 2.3. BSA Does Not Interfere with CK Loading and Release

**Figure 3:**
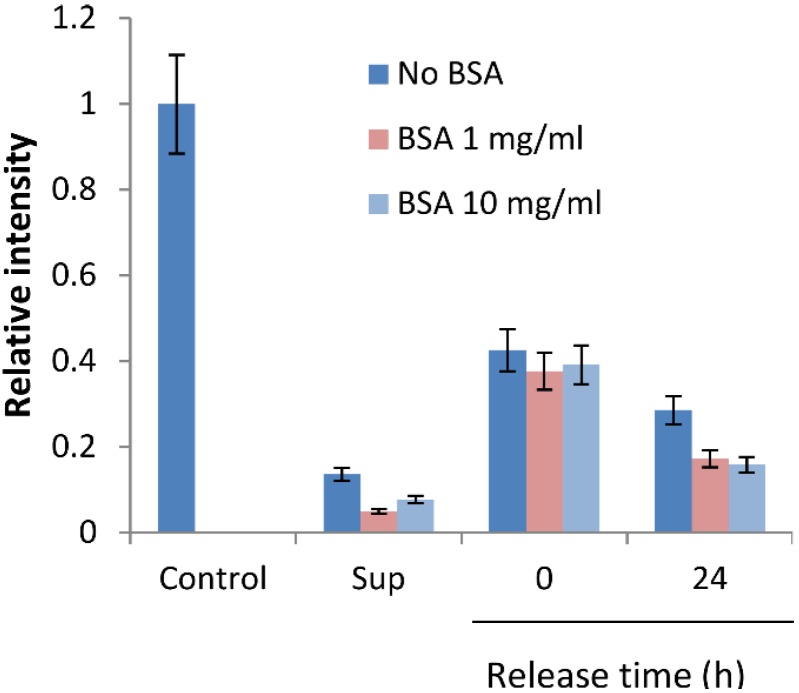
Effect of BSA on MIP-2 binding with and release from the Cibacron NPs. The CK (2 μg/mL) was loaded onto NPs (10% suspension) at 4 °C overnight in PBS buffer diluted 1:3 and supplemented with indicated concentrations of BSA in a total volume of 100 μL. After loading the particles were pelleted and Sups removed. The NP pellet was re-suspended in 1 mL of PBS with the indicated concentrations of BSA at room temperature. The amount of bound CK was determined by Western blot as described in Materials and Methods. The blot image was quantitated and relative intensities of the bands calculated. Error bars indicate SD calculated for three independent samples of control CK loaded on the same gel.

In the above experiments, at 37 °C, the binding buffer was supplemented with 1 mg/mL of BSA which is commonly used at concentration up to 10 mg/mL to decrease self-aggregation of proteins in solutions. However, BSA is potentially capable of interfering with the CK binding to bait dyes due to hydrophobic and electrostatic interactions. BSA is well known to bind a variety of proteins [37] and carries a negative charge of about 10 in neutral conditions [38]. On the other hand, the crosslinked structure of the particles is supposed to prevent BSA from entering the particle core [19], thus excluding a direct competition of BSA with the bait. To examine this, we tested the effect of BSA on the extent of CK binding and evaluated the CK dissociation rates in the presence of 1 and 10 mg/mL of BSA. Figure 3 shows that although BSA demonstrated a tendency to reduce the CK binding, its effect was not statistically significant. This suggests that the NP behavior in biological locations such as LNs would not be a subject of strong influence by the serum albumin as a major protein component of lymph.

### 2.4. Subcutaneous Injection Quickly Delivers NPs to Regional LNs

The behavior *in vivo* of the NPs we synthesized has not been previously characterized. Therefore, to ensure effective targeting of the LNs with the NP-bound CKs, it was important to demonstrate that the subcutaneously injected NPs would be delivered by the lymphatic drainage into the regional LNs. For this purpose the pNIPAm NPs co-polymerized with allylamine were covalently labeled with the Alexa Fluor^®^ 555 (Invitrogen, Waltham, MA, USA) fluorescent dye through a coupling reaction of the allylamine primary amino group with the succinimidyl ester-activated dye. The suspension of the fluorescent NPs in PBS was mixed with the equal volume of the 1% Evans Blue dye and injected into the hind footpads of mice. In preliminary experiments it was found that after 30 min the popliteal LNs became intensely stained blue and could be visually located for removal during surgery. The excised LNs were paraformaldehyde-fixed and paraffin-embedded for sectioning.

**Figure 4:**
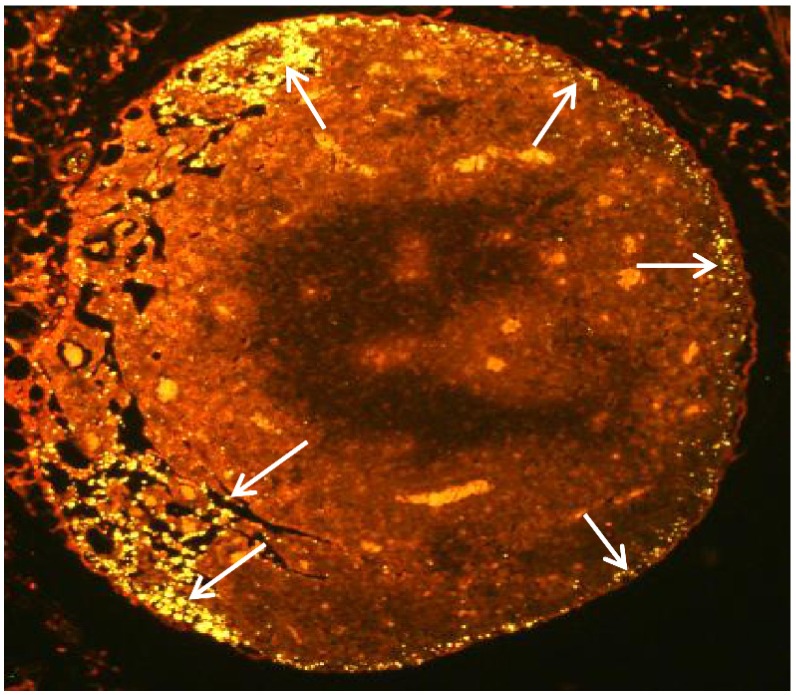
Fluorescent pNIPAm NPs labeled with Alexa Fluor 555 (yellow) quickly migrate to sub-capsular and medullar regions of popliteal LNs of mice (arrows). A suspension of NPs (20 μL) in PBS was injected into mouse hind foot pads for 30 min and the popliteal LNs surgically removed for histologic evaluation. The LNs were paraffin-embedded after fixation with paraformaldehyde, and the 8 μm tissue slices were mounted onto glass slide. The particles were observed at 555/570 nm using Olympus BX51 microscope with a TRITC filter set. Similar responses were detected in all three mice in the group challenged with CK-loaded NPs.

NPs from the periphery (such as the site of intradermal injection) are expected to quickly travel with the lymph flow to the LN *via* afferent lymphatic vessels entering at the convex side of the LN. Within the LN, the lymph drains through the subcapsular sinus, which is the space between the capsule and the inner part (cortex) of the LN. The lymph then flows inwards into trabecular sinuses, and finally into the medullary sinuses, before exiting through the efferent lymph vessels at the hilum on the concave side. Figure 4 shows a fluorescence microscopy image of the popliteal LN section after NP injection. The NPs were found to be well-dispersed and preferentially localized in the areas of subcapsular and medullar sinuses, while virtually absent from the trabecular sinuses in the cortical area. Similar results were observed in the more distant inguinal LNs (not shown). The subcapsular sinus localization favors the interaction of NPs with subcapsular macrophages and dendritic cells [39]. It is likely that the size of our NPs prevents them from flowing through the LN trabecular sinuses accessible to smaller NPs (<20 nm) [39].

### 2.5. CK-Loaded NPs Mobilize Immune Cells to the LNs upon Administration to Mice

**Figure 5:**
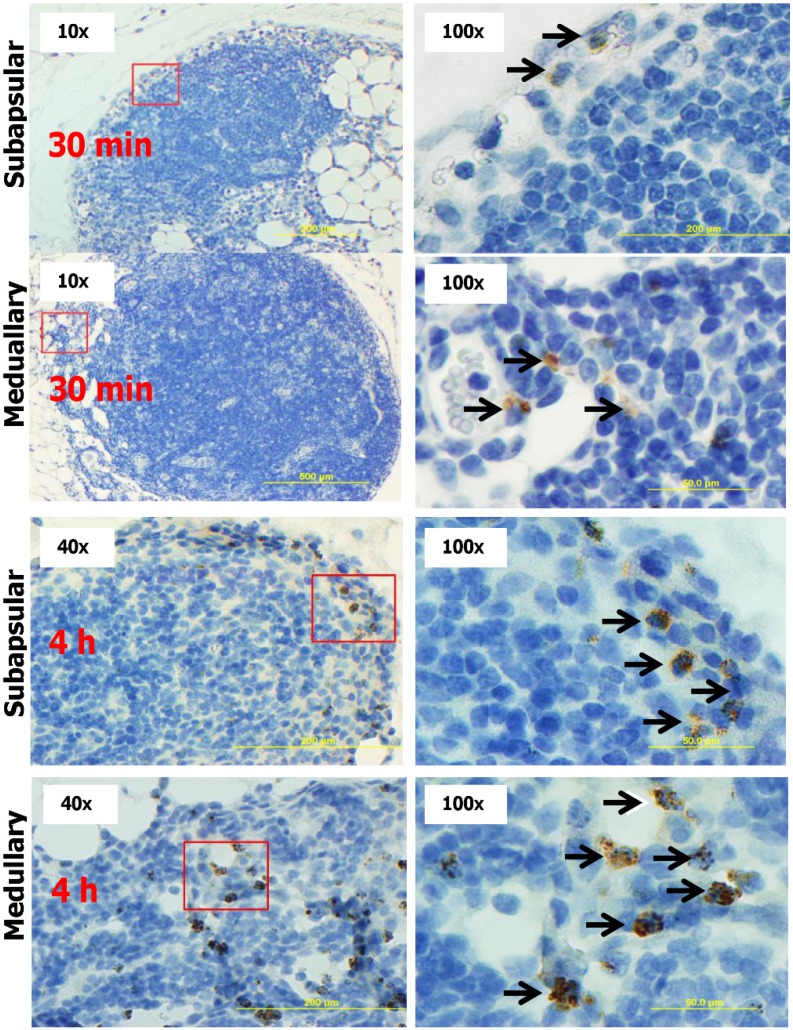
Representative images of the subcapsular and medullary regions of popliteal LNs after injection of Reactive Blue NP suspension (5% wet *v*/*v*, 50 µL) into each of the hind footpads of mice. H&E-stained sections after 30 min (**two top rows**) and 4 h (**two bottom rows**) post injection. Squared regions are shown on the right at higher magnification. Neutrophils (arrows) were immunostained brown for myeloperoxidase.

**Figure 6:**
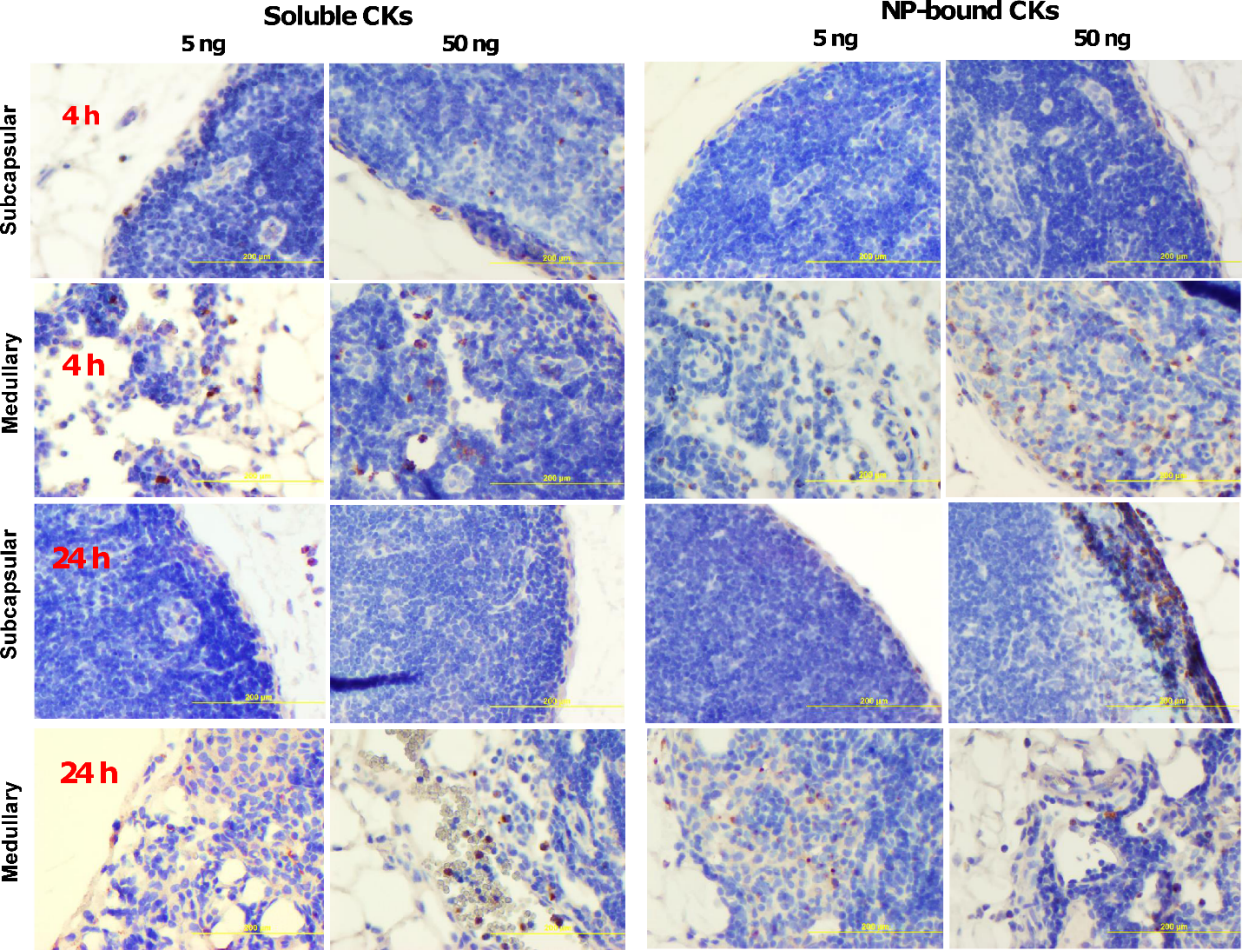
Representative images of the subcapsular and medullary regions of popliteal LNs after injection of the soluble (**two left columns**) and the Reactive Blue NP-loaded (**two right columns**) IL-8 and MIP-1α. H&E-stained sections after 4 h (**two top rows**) and 24 h (**two bottom rows**). The injected amount was 5 ng and 50 ng of each CK in the total volume of 50 µL. Neutrophils were immunostained brown for myeloperoxidase.

**Figure 7:**
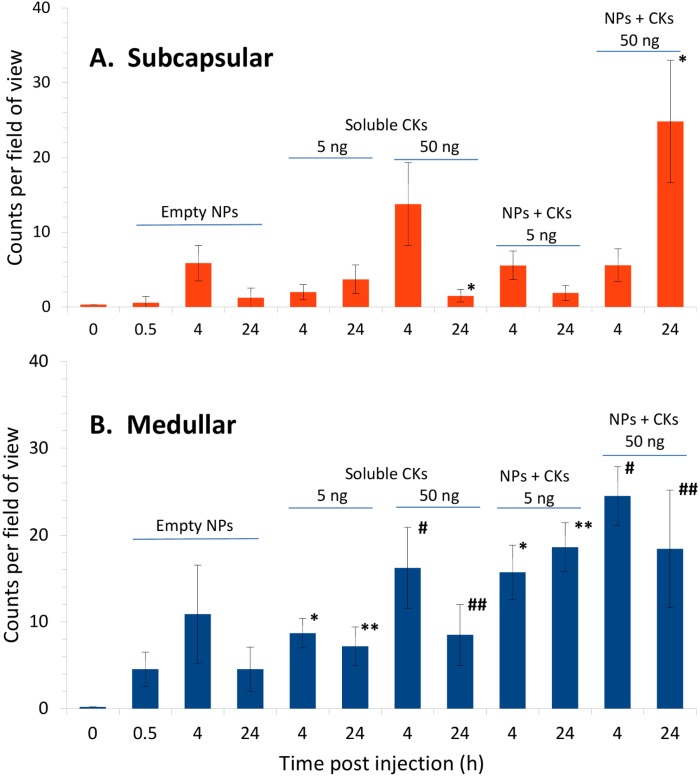
Enumeration of neutrophil counts in the subcapsular (**A**) and medullary (**B**) regions of popliteal LNs in the experiments corresponding to Figure 5 and Figure 6. Mice were injected with the soluble and the Reactive Blue NP-loaded IL-8 and MIP-1α. The number of myeloperoxidase-positive neutrophils in the tissues slices was counted under microscope in five randomly selected fields of view (0.02 mm^2^ each) for each of the indicated conditions (chemokine dosage, presence of NPs, and time post challenge). Error bars correspond to 95% confidence intervals. * and # indicate *p* ≤ 0.05 between the corresponding counts with and without NPs.

To demonstrate biological activity of CK-loaded NPs, we chose to test the combined activity of the neutrophil-attracting CKs (IL-8 and MIP-1α [40,41,42]). The neutrophils are major players during immune reactions demonstrating response to chemotactic stimuli within hours. These cells can be readily detected by immunohistochemistry due to the myeloperoxidase activity in their cytoplasmic granules. The NPs containing Reactive Blue bait were incubated with CKs to create concentrations of bound CKs of 1 and 0.1 μg/mL each. Small volumes (50 μL) of particle suspensions were injected into the hind footpads of mice. At certain times (30 min, 4 h, or 24 h) during post inoculation of the NPs, the mice received additional foot pad injections of the marker dye Evans Blue (1%). After 30 min the mice were euthanized, and the LNs (four per group) extracted for histological evaluation. Control mice received equal doses of the CKs as solutions without NPs or “empty” NPs without CKs. Representative images from these experiments are shown in Figure 5 and Figure 6 along with the quantitation of results in Figure 7.

Analysis of the LNs after administration of control NPs at 30 min and 4 h post injection showed a low number of migrated neutrophils in subcapsular and medullar regions (Figure 5), in agreement with the distribution pattern seen in the experiments with the fluorescent NPs. These early time points reflect the response of the first wave of neutrophils from a pool immediately available in the circulation [43]. This wave typically subsides after 24 h, and the mobilization of the additional number of cells from the bone marrow during the second wave of neutrophils takes place after several days [44]. As expected, the neutrophil counts (per microscope field of view under × 100 magnification) after the injection of empty NPs in the subcapsular region became reduced from 5.9 ± 2.4 at 4 h post infection to 1.2 ± 1.3 at 24 h, close to the level of naïve mice (0.3 ± 0.01) (Figure 7A). In the medullary region, the counts at 4 h decreased from 10.9 ± 5.6 to 4.6 ± 2.6 at 24 h (Figure 7B).

The injections of soluble CKs revealed a low-grade, dose-dependent neutrophil infiltration which was detectable at 4 h as well as 24 h post injection (Figure 6). Due to the small number of migrated cells (similar to the above experiments with empty NPs) the subcapsular space remained narrow without a substantial increase in the total cell density. We suggested that in the tested conditions the soluble CKs were unable to trigger a substantial neutrophil response as a result of fast dissipation of the bolus CKs’ doses. In support of this, at the highest soluble 50 ng dose the counts in the subcapsular region dropped from 13.8 ± 5.5 at 4 h to 1.5 ± 0.8 at 24 h (Figure 7A).

According to the above consideration, the early time points the NP-bound CKs were expected to display even less activity than the equal dose of the soluble one because of the smaller CK amount released by the NPs. In contrast, at longer times post injection we expected a sustained release of the CKs by the NPs to attract more neutrophils than the soluble CKs. A side-by-side comparison of the effects of soluble and NP-bound CKs confirmed the above considerations (Figure 7A). In the subcapsular region, the early 4-h response to the NP-bound CKs at both 5 ng and 50 ng doses did not demonstrate an increase over the level of empty NPs (5.9 ± 2.4). On the other hand, the response to the 50 ng soluble dose (13.8 ± 5.5) was substantially stronger than to the 5 ng dose (2.0 ± 1.0). However, at 24 h post injection the NPs loaded with a 50 ng CK dose demonstrated a widening of the subcapsular space associated with a massive accumulation of the leukocytes (stained blue), including the neutrophils (stained brown for MP) at the level of 24.8 ± 8.2, in sharp contrast with the soluble CKs (1.5 ± 0.8).

In comparison with the subcapsular region, the medullar one demonstrated overall higher levels of infiltrating neutrophils and increased sensitivity to CK stimulation (Figure 7B), likely reflecting their appearance from blood vessels, but not the afferent lymphatics [44]. The contribution of empty NPs was slightly elevated but remained low at the level of occasional cells. The NP-loaded CKs relative to the soluble CKs displayed their stimulating effect at lower dose of 5 ng (15.7 ± 3.2 *vs.* 8.7 ± 1.7) and sustained this effect until 24 h (18.6 ± 2.8 *vs.* 7.2 ± 2.2 at 5 ng and 18.4 ± 6.8 *vs.* 8.5 ± 3.5 at 50 ng).

## 3. Discussion

The NP materials capable of controlled release of biologically active substances hold promise for the development of new therapeutic approaches for a variety of diseases and adverse conditions. In each particular application, such materials are required to display a range of specific properties relevant to the capacity, stability, release rates, toxicity and other potential side effects, as well as the ease of manufacture. From this standpoint, the pNIPAm NPs represent an attractive platform offering a possibility to generate non-toxic, sufficiently stable, controlled-porosity hydrogel materials amenable to different chemical modifications. The incorporation of more than 20 different dye baits allowed us to modify the affinity of NPs towards the substances of interest for a variety of applications [19,21].

In this study, we explored the possibility of loading the CKs onto the dye-coupled NPs with the aim of creating the nanomaterials which would release their cargo in a controlled manner based on the affinity of a particular CK-bait pair. In contrast to the commonly used biodegradable NPs, the bait affinity NPs allow a possibility of CK loading in mild physiological conditions. In addition, the hydrogel matrix protects the loaded CK from proteolytic damage. The release rate can be adjusted by using different baits. While the exact nature of the dye interactions with target proteins is often unclear, the triazine dyes we used in this study as baits, such as Cibacron F3G-A or Reactive Blue 4, contain sulfate groups and condensed aromatic and heterocyclic hydrophobic rings, which are expected to mimic the naturally occurring sulfated glycoseaminoglycans such as the heparan and dermatan sulfates interacting with CKs in animal tissues [35,45,46]. We found that the affinities of tested CKs (IL-8 and MCP-1) to the bait-containing NPs were in range with the low-micromolar values reported in the case of endothelial cells [47].

The NPs pre-loaded with CK passively release their cargo (that is not covalently bound) according to the affinity off-rate thereby generating a source of free CK. The release process was tested for IL-8 and MIP-2 loaded onto Cibacron and Reactive Blue baits during an overnight incubation at 4 °C. To determine the dissociation rate the NPs were quickly pelleted, the supernatant removed, and the pellet was resuspended in the fresh portion of the buffer (PBS with or without BSA). At a certain time point, the NP were pelleted and the amount of CK remaining in the pellet was analyzed by Western blot in comparison with a control sample representing the CK concentration used for loading. As expected, the initial drop corresponding to the release of the loosely bound CK was followed by a slow phase typical for ligand diffusion out of the cross-linked polymer hydrogels [48]. The half-life times estimated from the data assuming the first-order dissociation reactions were found to be in the range of several hours (at 37 °C), in favorable agreement with dynamics of the immune cells responses to administered CKs. For example, the infiltration of neutrophils in response to intradermal administration of IL-8 to rabbits and mice peaks after 30 min and remains significant until 8 h [49,50,51]. MIP-1α activity is detectable in mice at 2 h after CK injection and peaks at 10 h. Monocyte, lymphocyte, and, to a lesser degree, eosinophil infiltration was observed peaking at 10–24 h [52]. Calculations show that 1 µL of our NPs will release the amount of CK sufficient to create physiological levels of CKs (1–100 nM [36]) in the volume of 1–100 mL.

An important feature of the pNIPAm hydrogel is the exclusion of large proteins from the inner part of the NPs [19]. As a result, the bait molecules within the NPs are accessible to CKs (having the molecular masses <20 kD), but not to the much bigger BSA. This allows avoiding the competition of CK with BSA for the bait during the process of the CK loading in the presence of BSA, frequently used to prevent aggregation and inactivation of CKs in solution. The same consideration explains our observation that the presence of BSA does not appear to have a substantial effect on the CK dissociation rate.

The pNIPAm NPs are known to be thermoreactive. They shrink upon temperature increase above the lower critical solution temperature (LCST) of 32 °C [53]. The LCST can differ depending on the ratio of hydrophilic and hydrophobic segments of the polymer. Although this topic was not investigated in the current study, our results indicate that the structural changes in the NPs caused by the shift from the ambient temperature of 25 °C to the animal body temperature of 37 °C did not impose considerable constraints on the dissociation of the CKs. The activation energy estimate for this process based on the data from the Table averages around 70 kJ/mol for chemical reactions of this type.

To prove the feasibility of our approach *in vivo*, we carried out animal experiments with the mixture of IL-8 (human CXCL8) and MIP-1α (murine CCL3) loaded onto Reactive Blue NPs. The combined action of these CKs was expected to display a synergistic effect. IL-8 is one of the major pro-inflammatory human CKs which direct migration of neutrophils. It is also highly active upon administration to mice [40]. IL-8 activates the CXCR1 and CXCR2 CK receptors of neutrophils [54,55,56] implicating them in development and promotion of tumor progression and numerous inflammatory disorders [57]. Modulation of the function of CXCR2 is considered as a possible therapeutic strategy in the treatment of inflammatory conditions in humans [57]. MIP-1α is crucial for immune responses towards infection and inflammation [58]. It activates human granulocytes (neutrophils, eosinophils and basophils) which can lead to acute neutrophilic inflammation. It also participates in the inflammatory response through the induction of synthesis and release of pro-inflammatory cytokines such as IL-1, IL-6 and TNF-α from fibroblasts and macrophages.

In response to chemotactic factors, the neutrophils migrate to the LNs after infections or vaccination [43,59,60,61]. Our results demonstrate a profound effect of NP-bound CKs on the neutrophil numbers in LNs, in contrast to the control NPs or the bolus injections of CKs, which caused only a mild infiltration of neutrophils. The timing of responses in comparison with controls indicates a sustained release of CKs maintaining a stimulation of migration until 24 h post injection. The differences we detected in the neutrophil counts between the subcapsular and medullar regions of LNs may reflect their migration though distinct pathways. The increased presence of neutrophils in the cortical (subcapsular) sinuses is consistent with the NP entry by way of afferent lymphatics while the medullar neutrophils likely originate from blood in the process of extravasation across vascular portals termed high endothelial venules [43,44]. The latter pathway is used by neutrophils in response to pathogen-mediated inflammation [62], supporting the possibility of their targeting by CK-releasing NPs during infectious disease.

Overall, our results establish feasibility of manipulation with the biological effects of CKs *in vivo* using pNIPAm NPs containing the chemically-coupled dyes as delivery vehicles reversibly binding and releasing the CK molecules. In future studies, we plan to apply this approach to the design of controlled release formulations that deliver natural soluble factors targeting different immune cells for antimicrobial and anticancer applications.

## 4. Materials and Methods

### 4.1. Materials

N-Isopropylacrylamide (NIPAm), N-N′-methylenebis(acrylamide) (BIS), potassium persulfate (KPS) and allylamine (AA), were purchased from Sigma-Aldrich (St. Louis, MI, USA), Cibacron Blue F3G-A was purchased from Polysciences, Inc. (Warrington, PA, USA). Unless specified otherwise, all other reagents were from Sigma-Aldrich, and were used as received. Water for all reactions, solution preparation, and polymer washing was distilled, further purified using a Millipore Milli-Q system to a resistance of 18 MΩ, and passed through a 0.2 μm nylon filter. Recombinant mouse CCL3 (MIP-1α), CCL2 (MCP-1), CXCL2 (MIP-2), and human CXCL8 (IL-8) were carrier-free from BioLegend (San Diego, CA, USA).

### 4.2. Synthesis of NPs

The NP synthesis was carried out essentially as described in [19]. pNIPAm particles with ~7% molar content of acrylic acid (AAc) relative to the total monomer were prepared via precipitation polymerization. NIPAm (9.0 g) and BIS (0.28 g) were dissolved in 250 mL of water, and the solution was then partially degassed by vacuum filtration through a 0.45 μm nylon filter. The filtered solution was purged with nitrogen at room temperature and a medium rate of stirring for 15 min, before AAc (0.5 g) was added to the reaction. Following the addition of AAc, the solution was purged with nitrogen for another 15 min and then heated to 75 °C. Once the reaction mixture had attained a stable temperature of 75 °C, polymerization was initiated with the addition of KPS (0.1 g) in 1.0 mL of water. The reaction was maintained at a constant temperature of 75 °C with stirring under nitrogen for 3 h. After this time, the reaction was allowed to cool to room temperature overnight with stirring under nitrogen. For the preparation of NIPAm functionalized with allylamine, the AAc was replaced with allylamine (670 µL, 12 µmoles). The particles were then harvested and washed by centrifugation for 20 min at 23 °C and 16,000 *g*, with the supernatant subsequently discarded. The pelleted particles were then re-suspended in 300 mL of water, and the suspended particles pelleted by centrifugation. This centrifugation-dispersion process was repeated a total of five times. Particles were stored as a suspension in water with a few drops of chloroform as an antimicrobial.

Cibacron Blue F3G-A (Cibacron) and Reactive Blue 4 (Reactive Blue), the reactive triazine dyes, were immobilized via direct reaction with the amine group of the allylamine units within the particles, displacing the lone chlorine on the disubstituted triazine ring of the dye [33]. Reactive Blue NPs were a kind gift from Ceres Nanoscience, Inc. Trypan Blue was coupled to the NPs by condensation of the amino group of the dye to the carboxylic group of acrylic acid present in the pNIPAm-co-AAc NPs using activation with *N*-(3-dimethylaminopropyl)-*N*′-ethyl carbodiimide as described [27]. After the incorporation of the dyes, the particles were harvested and washed in water by five cycles of centrifugation-dispersion for 20 min at 23 °C and 16,000 *g*, with the supernatants discarded. Finally, NPs were re-suspended in water with a few drops of chloroform as antibacterial agent. To demonstrate the absence of bacterial contamination, 100 μL of particle suspension were plated on the Luria broth agar and incubated for up to 48 h at 37 °C. The N4 Plus PCS Submicron Particle Analyzer (Beckman Coulter, Brea, CA, USA) was used to determine the particle size and polydispersity index.

### 4.3. Analysis of CK Binding Using ELISA and Western Blot

The sandwich ELISA Ready-SET-Go!^®^ IL-8 and MCP-1 kits (eBioscience, San Diego, CA, USA) were used according to the manufacturer’s protocols to measure the IL-8 and MCP-1 binding to the Cibacron and Reactive Blue NPs. The CKs supplied with the kits were diluted with PBS to concentrations of 0.25–1.0 ng/mL and mixed with equal volumes of NPs washed 3× with PBS by pelleting the NPs at 16,000 *g* for 5–10 min and re-suspending the pellet in a fresh portion of PBS. Different dilutions of the stock suspension (5% wet pellet *v*/*v*) were used. The mixtures of NPs with CKs were incubated for indicated periods of time at 4 °C, the NPs were pelleted at 16,000 *g* for 5–10 min at room temperature, and the Sups were withdrawn for analyses. Triplicate wells in 96-well plates were used for Sups and control dilutions of the standard CKs.

For Western blot analysis, the samples of Sups and pelleted NPs were mixed with standard 2× SDS-PAGE loading buffer (Invitrogen), boiled for 5 min and loaded on 4%–20% polyacrylamide gel. Protein bands from the gel were transferred onto a nitrocellulose membrane using iBlot Gel Transfer Device (Invitrogen) and probed with the CK-specific polyclonal antibodies (from Biolegend for MIP-2 and LifeTechnologies for IL-8) followed by the appropriate secondary antibodies conjugated with horseradish peroxidase. SuperSignal West Femto Maximum Sensitivity Substrate (Pierce) was used to generate chemo-luminescence of the protein bands, which was measured with a Molecular Imager ChemiDoc XRS System (Bio-Rad, CA, USA). The relative intensities of bands were calculated after densitometry using the QuantityOne 4.6.5 software (Bio-Rad). According to control measurements with different amounts of CKs tested in triplicates within the same experiment, the standard deviations (SD) of relative band intensities were in the range of 7%–17% (*n* = 3).

### 4.4. Labeling of pNIPAm-co-AA Particles with Alexa Fluor 555

Alexa Fluor 555 (Invitrogen) is a bright orange dye widely used in fluorescent imaging. It is water-soluble and pH-insensitive from pH 4–10. The succinimidyl ester of Alexa Fluor^®^ 555 was used for conjugating the dye to primary amines on pNIPAm-co-AA NPs. For this purpose, 100 μL of NPs were washed 2× with 1 mL of 50 mM bicarbonate buffer, pH 8.3, re-suspended in 500 μL of the buffer, and mixed with 50 μL of the dye solution (1 mg in 100 μL of DMF). After 1 h at room temperature, the particles were washed 3× with 1 mL of PBS (pH 7.4) and finally re-suspended in 500 μL of PBS. The particles were observed at 555/570 nm using Olympus BX51 microscope with a TRITC filter set. The number of labeled NPs was counted after appropriate dilution and was found to be about 7 × 10^5^ per 1 μL of original suspension. The particles suspended in water had an average size of 520 ± 54 nm with a dispersion index of 1.0 ± 0.3 indicating a slight tendency to aggregation. Overall, the particles were well-dispersed and migrated readily through the lymphatics. For injection into the hind leg footpads of mice, the NP suspension was mixed with equal volume of 2% tracer dye Evans Blue in PBS. This dye allowed us to locate the LNs during surgery and did not quench the fluorescence of Alexa Fluor 555. The LNs were prepared as described below and the particles were observed as described above.

### 4.5. Animal Challenge and LN Analysis

All animal procedures were approved by the George Mason University’s Institutional Animal Care and Use Committee. Female 6–8-week-old DBA/2 mice (Jackson Labs) received food and water *ad libitum* and were challenged into both hind footpads with 50 μL of Reactive Blue NP suspensions or control CK solutions, three animals per challenge group. The NPs (5% wet *v*/*v*) suspension contained either 0.1 ng/μL or 1 ng/μL of each IL-8 and MIP-1α. Control solutions contained the same amounts of CKs without NPs, or the NPs without CKs. The mixtures were incubated for 18 h at 4 °C and then used for animal inoculations. Groups of mice were euthanized at 30 min, 4 h, and 24 h post inoculation. Thirty min before euthanasia, the animals were anesthetized with ketamine/xylazine and 20 μL of 1% tracer dye Evans Blue in PBS were injected into foot pads. In some experiments, the dye solution contained fluorescent nanoparticles. The LNs were surgically removed into 10% neutral buffered formalin solution for histological evaluation. After fixing in formalin, the tissues were embedded in paraffin, the paraffin blocks were sliced into 8 μm sections, and mounted onto glass slides for standard hematoxylin/eosine (H&E) staining and further microscopic evaluation. To detect the presence of neutrophils, sections after antigen retrieval were incubated in 3% hydrogen peroxide in methanol for 5 min to inhibit peroxidase activity, blocked with Dako Protein block (Dako) for 5 min, and then incubated with a primary anti-myeloperoxidase antibody (Ab9535 from AbCam, dilution 1:50) for 30 min, followed by Dako anti-rabbit EnVision+ HRP-Labeled Polymer (Dako). Colorimetric detection was completed with diaminobenzidine for 5 min, and slides were counterstained with hematoxylin.

## 5. Conclusions

We propose an *in vivo* CK gradient remodeling approach based on sustained release of CKs with a half-life of several hours at 37 °C by the crosslinked hydrogel open meshwork NPs containing internal covalently linked dye affinity baits for a reversible CK binding. The NPs loaded with IL-8 and MIP-1α demonstrated increased neutrophil recruitment to LNs of mice after footpad injection while mice injected with control NPs without CKs or bolus doses of soluble CKs alone showed only a marginal neutrophil response. This technology provides a new means to therapeutically direct or restore immune cell traffic, and can also be employed for simultaneous therapy delivery.
